#  Hereditary Breast-Ovarian Cancer Syndrome in Russia 

**Published:** 2010

**Authors:** A.P. Sokolenko, A.G. Iyevleva, N.V. Mitiushkina, E.N. Suspitsin, E.`V. Preobrazhenskaya, E.Sh. Kuligina, D.A. Voskresenskiy, O.S. Lobeiko, N.Yu. Krylova, T.V. Gorodnova, K.G. Buslov, E.M. Bit-Sava, G.D. Dolmatov, N.V. Porhanova, I.S. Polyakov, S.N. Abysheva, A.S. Katanugina, D.V. Baholdin, G.A. Yanus, A.V. Togo, V.M. Moiseyenko, S.Ya. Maximov, V.F. Semiglazov, E.N. Imyanitov

**Affiliations:** Petrov Institute of Oncology, St. Petersburg; State Pediatric Medical Academy, St. Petersburg; Medical Academy for Postgraduate Studies, St. Petersburg; City Oncological Hospital, St. Petersburg; Kuban State Medical University, Krasnodar

**Keywords:** breast cancer, ovarian cancer, hereditary cancer syndromes, *BRCA1, CHEK2*, NBS

## Abstract

Hereditary breast-ovarian cancer syndrome contributes to as much as 5–7% of breast cancer (BC) and 10–15% of ovarian cancer (OC) incidence. Mutations in the “canonical” genes*BRCA1*and*BRCA2*occur in 20–30% of affected pedigrees. In addition to*BRCA1*and*BRCA2 *mutations, germ-line lesions in the*CHEK2*,*NBS1*, and*PALB2*genes also contribute to familial BC clustering. The epidemiology of hereditary breast-ovarian cancer in Russia has some specific features. The impact of the “founder” effect is surprisingly remarkable: a single mutation,*BRCA1*5382insC, accounts for the vast majority of*BRCA1*defects across the country. In addition, there are two other recurrent*BRCA1*alleles:*BRCA1*4153delA and*BRCA1*185delAG. Besides*BRCA1*, in Russia breast cancer is often caused by germ-line alterations in the*CHEK2*and*NBS1*genes. In contrast to*BRCA1*and*BRCA2*, the*CHEK2*and*NBS1*heterozygosity does not significantly increase the OC risk. Several Russian breast cancer clinics recently started to investigate the efficacy of cisplatin in the therapy of*BRCA1*-related cancers; initial results show a unique sensitivity of*BRCA1*-associated tumours to this compound.

##  Introduction 

Breast cancer (BC) and ovarian cancer (OC) contribute significantly to cancer incidence and mortality. BC is the most frequent malignant pathology in women, with the lifetime risk reaching approximately 10%. In some cases, BC can be easily diagnosed at early stages and ultimately cured. Unfortunately, even with the implementation of total population screening, the BC related mortality rate has not decreased significantly, due to insufficient sensitivity of available diagnostic methods, as well as the high metastatic potential of some BC forms [1]. OC is a much rarer disease than BC, being found only in 1.5% of women around the world; however, it is almost always diagnosed at late (incurable) stages. Early ovarian tumours do not cause symptoms and are often missed by ultrasound examination and СА-125 biomarker assay [2]. Both BC and OC are diseases of the reproductive system; therefore, their hormonal, metabolic, and behavioural risk factors are common to a certain extent. Interestingly, these two diseases are the main components of the most frequent genetic disease – hereditary breast-ovarian cancer (HBOC) syndrome [3].

HBOC has been intensively studied since the early 1990s. In 1994, the first gene associated with this syndrome was discovered and named *BRCA1* (BReast CAncer 1) [4], and the second gene, *BRCA2* , was discovered a year later [5]. Although *BRCA1* and *BRCA2* code for different proteins, their products play a key role in preserving genome integrity by participating in DNA repair [6]. Notably, *BRCA1* or *BRCA2* mutations occur only in 20–30% of familial BC/OC cases. There has been an active search for other hereditary BC/OC genes. The effort has already helped to identify several new relevant genes, e.g. *CHEK2* , *NBS1* , *PALB2* etc. [7].

The first studies on the contribution of the *BRCA1* and *BRCA2* genes in BC and OC incidence were performed on European and North American women. The mutations in these genes are very diverse [8], which complicates *BRCA* diagnostics. Indeed, to perform the complete analysis of *BRCA1* and *BRCA2* , one needs not only to perform full sequencing of these long genes, but also to find rearrangements using the MPLA method (multiplex ligation-dependent probe amplification). In the mid-1990s, it was established that the so-called “founder effect” was present in some small isolated ethnic groups. For example, in females Ashkenazi Jew nearly all *BRCA1* and *BRCA2* mutations are represented by only 3 recurrent alleles, i.e. *BRCA1* 185delAG, *BRCA1* 5382insC, *BRCA2* 6174delT; *BRCA2 * 999del5 is a prevailing mutation in Icelandic females [9, 10]. Population-specific distribution of hereditary cancer mutations may significantly affect the design of genetic studies. In countries without a strong founder effect, only cancer cases with a high probability of detecting the mutation are usually taken into the analysis; they include oncological patients with a proven cancer history in their family and/or patients with multiple primary tumours and/or young women with BC or OC. The presence of the founder effect greatly simplifies the DNA testing procedure, enabling comprehensive studies, such as revealing the influence of hereditary cancer gene mutations on the overall BC/OC incidence rate, as well as analyzing the gene mutations in healthy women [11].

## Epidemiology of the *BRCA1* , *BRCA2* , *CHEK2* and *NBS1* mutations in Russia

In Russia, the studies of the HBOC syndrome were initiated later than in the U.S. and Europe but they produced rather unexpected results. The first paper published in 1997 reported on the results obtained in patients with familial OC living in Moscow and several other regions of the former Soviet Union [12], the main result being the extremely high frequency of the *BRCA1* 5382insC mutation. As was mentioned above, this mutation had been first found in Jewish women; therefore, it had been for many years considered in the context of that particular ethnic group [13]. However, it appeared that the *BRCA1* 5382insC mutation was not of Jewish origin. This mutation is found not only in females living in various regions of Russia, but also in native populations of Poland, Lithuania, Latvia, and Belarus [14–17]. It is perhaps more accurate to say that the *BRCA1* 5382insC mutation is of Slavic origin, and that the relatively high frequency of this mutation in Ashkenazi Jews observed mostly in Eastern Europe is likely due to the long coexistence of the Slavic and Jewish peoples in the Baltic region and adjacent territories.

The epidemiology of the *BRCA1* 5382insC mutation is surprising, to say the least, since it contradicts the stereotype of the multinational culture in the Russian Empire and the Soviet Union. *BRCA1* 5382insC accounts for up to 90% of all *BRCA1* mutations in women living in distant regions of Russia, ranging from Moscow to St. Petersburg, Krasnodar, Tomsk, etc. [12, 18–20, 22–24, 26]. Moreover, this mutation is dominant in neighboring countries with a mostly Slavic population such as Poland, Belarus, Latvia, and Lithuania [14–17]. Notably, the relative genetic homogeneity of the Slavs is in accordance with the results of general population studies on the genetic diversity of people living in Russia [31]. The *BRCA1* 5382insC allele frequency in healthy women is approximately 0.1%. This variant accounts for approximately 2–5% of total BC cancer incidence. Among the high-risk patients (familial cancers, bilateral breast tumours, or early onset cancers), this mutation is observed in 10% of patients. The *BRCA1* 5382insC contribution to the OC rate is even bigger: this mutation is found in 10–15% of patients (Table). It is important to note that in contrast to the BC, the *BRCA1* 5382insC distribution in women with OC is independent of age, family history, and the number of primary tumours [26]. Therefore, while DNA testing of BC patients can be restricted by high-risk cases, all OC patients have to undergo BRCA testing.

In the pioneering report [12], the relatively frequent *BRCA1* 4153delA (4154delA) mutation was described. The mutation was found not only in Russian patients, but also in those from other neighboring Slavic countries [14–17]. The *BRCA1* 4153delA frequency in Russian patients, however, is an order of magnitude lower than that of the *BRCA1* 5382insC mutation, which complicates the study of the *BRCA1* 4153delA epidemiology. Polish scientists had reported on the preferential association of *BRCA1* 4153delA with OC [14, 32]; however, their observations could not be confirmed in later studies [21].

A number of Russian studies indicate that there is a relatively high frequency of the “Jewish” *BRCA1* 185delAG allele in Russian patients [20, 23, 24, 26]. In contrast to the *BRCA1* 5382insC mutation, however, this mutation is not dominant and could be better explained by interethnic marriages.

The * BRCA1* gene mutations in familial BC/OC patients have been repeatedly analyzed by sequencing of all coding sections, with similar results obtained in Moscow, St. Petersburg, and Tomsk. It has been shown that non-founder mutations are much rarer in Russia than in Europe and North America [12, 18–20, 23, 33]. Given the rapidly falling costs of DNA analysis, it is logical to expect an increase in the use of *BRCA1* sequencing even for the patients with low probability of cancer genetic predisposition. So far, there has been only one study on gross rearrangements of the *BRCA1* gene, and the data indicate a low frequency of such mutations in Russian patients with hereditary BC/OC [33].

While the *BRCA1* gene has been systematically analyzed, data on the *BRCA2* mutations in Russia are scarce. In Siberia, there have been several reported cases of *BRCA2* being inactivated in hereditary BC/OC patients [18]; at the same time, studies performed in Moscow revealed no connection between this gene and hereditary BC/OR in the European part of Russia [23]. Polish scientists performed comprehensive studies showing that the *BRCA2* mutations contributed very little to BC and OC aetiology in Slavs [34].

**Table 1:** Hereditary breast-ovarian cancer genes in Russia

Gene	Major mutations	Frequency in healthy subjects	Frequency in “high-risk” (familial and/or bilateral and/or young-onset) breast cancer patients	Frequency in non-selected breast cancer patients	Frequency in ovarian cancer patients	References
BRCA1	5382insC, 4153delA, 185delAG	~ 0.1%	~ 10%	2–4%	> 10%	[18–26]
CHEK2	1100delC, IVS2+1G>A	< 1%	~ 5%	~ 2%	<1%	[26–29]
NBS1	657del5	0.5%	~ 1%	0.7%	<1%	[29, 30]

Another interesting feature of Russian patients is the frequent occurrence of *CHEK2* mutations. This gene, as *BRCA1* and *BRCA2* , participates in the maintaining of genomic integrity. Heterozygous *CHEK2* mutations are frequent in Finland, the Netherlands, Poland, and several other countries [14, 35]. In Russia, *CHEK2* mutations are found in fewer than 2% of “random” BC patients, and in up to 5% of hereditary cancer patients [27]. In contrast to the situation with *BRCA1* and *BRCA2,* heterozygous inactivation of *CHEK2* does not increase the risk of OC [21, 26].

Another important gene for Russia is *NBS1* ( *NBN* ). Homozygous defects of this gene were found in patients with serious immunodeficiency, the so-called Nijmegen breakage syndrome [36]. Heterozygous *NBS1* mutations are observed mostly in Slavs, and they are associated with an increased BC risk [30, 37, 38]. No increased frequency of this gene defect is observed in OC patients [26]. Nevertheless, in the only reported case of combined germ-line heterozygosity for *BRCA1* and *NBS1* genes in ovarian tumor, there was somatic inactivation of the *NBS1* gene, whereas the *BRCA1* gene remained intact [39]. This observation may be an argument speaking in favor of the involvement of the *NBS1* gene in the degree of OC risk.

##  Medical aspects of hereditary BC and OC in Russia 

The main goal of hereditary cancer syndrome diagnostics is to find healthy women with corresponding mutations. It is believed that timely detection of the genetic defect can help to avoid fatal cancer outcome. Women with heterozygous *BRCA1* and *BRCA2* genes are under regular observation for early BC/OC diagnostics. In addition, preventive surgical removal of target tissues is recommended [40] to women with a BRC A mutation [40].

Healthy carriers of hereditary cancer genes are usually found during the examination of relatives of BC or OC patients with the genetic defect. According to current ethical standards, the patient herself should encourage her relatives to undergo DNA analysis. Our experience shows that very few relatives of the *BRCA* mutation carriers undergo DNA testing *.* This could be because either hereditary cancer patients conceal their condition to their relatives or the healthy women avoid medical procedures aimed at determining cancer risk. Even more surprising is the fact that the majority of healthy women with *BRCA1* mutations monitored by us are extremely careless when it comes to undergoing preventive screening. Preventive surgery presents the biggest challenge. While it has become a routine clinical practice in the U.S., Canada, Western and Eastern Europe, Israel, Australia, South Africa, Japan, Korea and other countries, yet in Russia the discussion of such an option is suppressed or distorted not only by ordinary people but even by the medical community.

While preventive measures for BRCA carriers are frequently neglected, many doctors are enthusiastic to try novel therapeutic schemes for HBOC patients. In 2009, Polish scientists published the results of clinical studies showing the unique sensitivity of *BRCA1* -associated tumors to cisplatin [41]. This is possible because of unique therapeutic window. In tumors of the *BRCA1* mutation carriers, complete inactivation of this gene is observed. It causes a homologous recombination defect. *BRCA1* -deficient cells are extremely vulnerable to cisplatin, a well-known DNA crosslinking compound causing double-strand breaks. It is important that normal tissues, in contrast to neoplasms, retain heterozygous *BRCA1* status, the presence of a single functional copy of the gene being sufficient for performing its functions. Russian scientists were the first to provide independent confirmation of the results of Byrski *et al* . [42]. Cisplatin is now commonly used for the therapy of *BRCA1* -associated tumors in several Russian clinics.
